# Tumor of the Neck Treated by the Use of the Knife, Followed by Death

**Published:** 1893-01

**Authors:** F. S. Davis

**Affiliations:** Quincy, Mass.


### INTERNATIONAL HAHNEMANNIAN ASSOCIATION MEETING OF 1892.

### TUMOR OF THE NECK TREATED BY THE USE OF THE KNIFE, FOLLOWED BY DEATH.

### TWO SIMILAR CASES TREATED WITH REMEDIES AND BOTH CURED.

F. S. Davis, M. D., Quincy, Mass.

Case 1.—Mr. J. M. H., æt. eighty-seven. A very well preserved man, above medium height; large frame and of average weight; dark eyes; hair formerly dark, now white; occupation, shoemaker. Consulted me December 23d, 1891, for treatment of a swelling on right side of neck just above the clavicle and directly over the large blood-vessels.

The tumor was about two inches through at base, was quite soft, and appeared filled with fluid. It was painful, a beating being constantly felt in it. I advised the use of remedies, but he would not stop short of having it opened, so he might be done with the sore at once. He consulted Dr. Horace Packard at the Massachusetts Homœopathic Hospital, who drew from the tumor quite a quantity of fluid for examination and set a day for operation—January 2d, 1892. On that day the tumor was opened by directing through the apex and opening two inches long to the base of tumor. A yellowish fluid escaped, exposing the deep-lying jugular. A little gauze was pressed into the opening, and the patient returned to Quincy, where he died of pyæmia January 25th. The wound never gave any signs of healing, remaining open and discharging a thin fluid of a peculiar musty odor. There was no relief for the pain; it was rather increased.

Case 2.—Miss J. B., of Boston. A short, thick-set woman, æt. forty-three. Dark hair and eyes; who had enjoyed quite good health for years.

Was in charge of laundry work in a hotel. Her duties kept her in the basement of building, and it was often more or less damp there and quite cool most of the time, as no sun came into the room. States that two weeks ago noticed a swelling on left side of neck quite low down and just in line downward from the ear. The swelling was *hard* and *painless*. Consulted Dr. Richardson, of Boston, who *painted* it and also advised a poultice, which was used for several days. Dr. R. got a room at the hospital, and was to open the swelling when sufficiently softened.

By a friend she was advised to consult me if she wanted to be cured without the knife.

She called on me February 26th, and gave me the history of her case, adding that just before the swelling begun she had an attack of nausea with chill and vomiting of bile; bowels irregular and constipated. She said she thought she might have got cold working in the *cold and dampness*. I gave one dose of Dulc.$^{200}$, and the swelling gradually disappeared, and there has been no trouble since.

CASE 3.—Michael B., æt. thirteen months, was brought to me April 24th, 1892, with an abscess forming on left side of neck below left ear. It was a little soft at apex, not much painful, about two inches in diameter.

The child presented a skin with disposition to suppurate wherever scratched, and, at *roots of nails*, there were little nodules under the skin about neck and of the scalp about neck.

The mother of the child feared the abscess would have to be cut and wanted to poultice it. This I did not permit.

One dose of Sil.$^{200}$ cured the case, bringing the abscess to a head and opening without the knife, and improved the health.

I have reported these cases because, while treating them, I was impressed with the importance of assisting nature with homœopathic treatment rather than interfering with her efforts by using the knife.

That there are cases calling for surgical treatment no one will deny, but I believe it is our duty to insist that wherever it is possible to do so, the indicated remedy must be given a sufficient time to test its powers to help every case.

By *treating* the *patient* rather than the outward manifestations of disease we may most surely meet with success.

The use of the knife never can remove from the patient any disease; it never can give new force to the efforts of nature. The vital principle is not strengthened by its use, neither is the constitution fortified against any disease. Why should it be used until the more certain and deep-acting homœopathic remedy has failed to restore the vital-force to its natural harmonious action?

### Discussion.

Dr. Long—In the third case was the tumor anterior or posterior to the ear?

Dr. Davis—Directly under the ear.

Dr. Dever—Dr. Davis's paper confirms the fact that homœopathic remedies will cure cases considered surgical, but it does not settle the question when the time for remedies ceases and the time for surgery begins. There are many cases which come to us which have been allowed to drift into an incurable state. A homœopathic physician has not been called in, and the case has been neglected, the vital-force has been exhausted by disease, and it is then necessary to use surgical interference. It is true that we draw a dividing line at a different place from the rest of our school, but still we have a place for it. The tendency of the discussion so far would lead an outsider to the conclusion that surgery was never to be employed.

Dr. Fincke—I am sorry that the paper of Dr. Stow was not read, because it treated on that very point. I wish he was here to read it, for that is a difficult point to decide upon.

Dr. Winn—I saw the case that Dr. Davis writes of at the time of the operation. Dr. Packard opened the sac, and a clear fluid-like serum came out. He found some very deep sinuses, and concluded it would be ill-advised to do anything further. The whole operation was trivial; it was done by chilling the surface and puncturing. I cannot see any points of comparison between the two cases, and think the operation had nothing to do with the fatal termination of the case.

Dr. Davis—I have simply to say that in reporting these cases I was lead by the results of observation on the case before and during the operations. The patient was aged and feeble at best, and would not live many years, perhaps, under any circumstances. I thought that by using the remedies indicated by his constitutional peculiarities I might do better than by an operation.

